# Remotely Delivered Interventions to Support Women With Symptoms of Anxiety in Pregnancy: Mixed Methods Systematic Review and Meta-analysis

**DOI:** 10.2196/28093

**Published:** 2022-02-15

**Authors:** Kerry Evans, Stefan Rennick-Egglestone, Serena Cox, Yvonne Kuipers, Helen Spiby

**Affiliations:** 1 School of Health Sciences University of Nottingham Nottingham United Kingdom; 2 Institute of Mental Health School of Health Sciences University of Nottingham Nottingham United Kingdom; 3 School of Health and Social Care Edinburgh Napier University Edinburgh United Kingdom

**Keywords:** anxiety, pregnancy, antenatal, systematic review, digital interventions, eHealth, remote interventions, electronic health, parenting, remote delivery, therapy, CBT, fear, distress, mobile phone

## Abstract

**Background:**

Symptoms of anxiety are common in pregnancy, with severe symptoms associated with negative outcomes for women and babies. Low-level psychological therapy is recommended for women with mild to moderate anxiety, with the aim of preventing an escalation of symptoms and providing coping strategies. Remotely delivered interventions have been suggested to improve access to treatment and support and provide a cost-effective, flexible, and timely solution.

**Objective:**

This study identifies and evaluates remotely delivered, digital, or web-based interventions to support women with symptoms of anxiety during pregnancy.

**Methods:**

This mixed methods systematic review followed a convergent segregated approach to synthesize qualitative and quantitative data. The ACM Digital Library, Allied and Complementary Medicine Database, Applied Social Sciences Index and Abstracts, Centre for Reviews and Dissemination database, the Cochrane Central Register of Controlled Trials, the Cochrane Library, CINAHL, Embase, Health Technology Assessment Library, IEEE Xplore, Joanna Briggs Institute, Maternity and Infant Care, MEDLINE, PsycINFO, and the Social Science Citation Index were searched in October 2020. Quantitative or qualitative primary research that included pregnant women and evaluated remotely delivered interventions reporting measures of anxiety, fear, stress, distress, women’s views, and opinions were included.

**Results:**

Overall, 3 qualitative studies and 14 quantitative studies were included. Populations included a general antenatal population and pregnant women having anxiety and depression, fear of childbirth, insomnia, and preterm labor. Interventions included cognitive behavioral therapy, problem solving, mindfulness, and educational designs. Most interventions were delivered via web-based platforms, and 62% (8/13) included direct contact from trained therapists or coaches. A meta-analysis of the quantitative data found internet-based cognitive behavioral therapy and facilitated interventions showed a beneficial effect in relation to the reduction of anxiety scores (standardized mean difference −0.49, 95% CI −0.75 to −0.22; standardized mean difference −0.48, 95% CI −0.75 to −0.22). Due to limitations in the amount of available data and study quality, the findings should be interpreted with caution. Synthesized findings found some evidence to suggest that interventions are more effective when women maintain regular participation which may be enhanced by providing regular contact with therapists or peer support, appropriate targeting of interventions involving components of relaxation and cognitive-based skills, and providing sufficient sessions to develop new skills without being too time consuming.

**Conclusions:**

There is limited evidence to suggest that women who are pregnant may benefit from remotely delivered interventions. Components of interventions that may improve the effectiveness and acceptability of remotely delivered interventions included providing web-based contact with a therapist, health care professional, or peer community. Women may be more motivated to complete interventions that are perceived as relevant or tailored to their needs. Remote interventions may also provide women with greater anonymity to help them feel more confident in disclosing their symptoms.

## Introduction

Symptoms of anxiety are common in pregnancy and have been reported across all 3 trimesters [1,2]. Recent reviews have reported worldwide prevalence rates of 15% to 20% in the antenatal period, with higher rates reported in low- to middle-income countries, particularly among poorer women with gender-based or psychiatric risks [3-5]. Risk factors for developing anxiety during pregnancy include demographic and socioeconomic factors (lack of partner support, young age, lower education, smoking, and being overweight), psychological factors (past mental health concerns, reduced emotional well-being, stressful events, low self-esteem, and negative coping styles), and obstetric factors (previous pregnancy loss and complications in pregnancy) [6,7]. Antenatal anxiety is reported to be associated with postpartum depression, reduced rates of breastfeeding, prematurity, and preterm birth [8,9].

Facilitated self-help and low-intensity psychological interventions are recommended as the first-line treatment option in pregnancy within a stepped care approach [10]; however, there are limited data on the effectiveness of interventions for the antenatal period [11]. Vigod and Dennis [12] discussed that interventions delivered via the internet might present a solution for overcoming the barriers of access to treatment for perinatal mental health disorders, such as a lack of specialist psychological and psychiatric support, the cost of services and transportation, and childcare requirements. Interventions can be delivered as unguided resources to support or replace patient–provider interactions or as guided interventions that may include live interactions over telephone or video or contact with therapists using digital messaging. Web-based interventions and peer discussion groups may benefit women by reducing the stigma of mental illness in pregnancy, addressing treatment barriers, and strengthening social support mechanisms. Web-based therapist-assisted interventions may offer women flexibility and convenience and be more efficient than the delivery of one-on-one therapy [12]. In particular, the use of mobile technology is thought to offer a low-cost solution for improving treatment accessibility and sustainability. In high-income countries, between 84% and 99% of people aged <35 years own a smartphone, which would include women in their prime reproductive years [13,14].

A recent review of internet-delivered psychological interventions for perinatal anxiety and depression [15] included 2 cognitive behavioral therapy (CBT) interventions for women with fear of childbirth [16] and symptoms of anxiety and depression [17]. Improvement in symptoms was reported, and participant satisfaction was positive in both studies. A scoping review of mobile health apps and text-based interventions for perinatal anxiety and depression (n=26 publications from 22 studies) [14] identified 1 intervention focused solely on anxiety and 9 interventions for both depression and anxiety. Intervention strategies included peer support, psychoeducation, and active therapy.

Anxiety symptom screening for maternity care, which, in the United Kingdom, uses a simple 2-item measure (Generalized Anxiety Disorder [GAD] 2 items) [18], is recommended to identify where women may need further support or referral for specific diagnosis by specialist mental health professionals. Locating interventions that can be integrated within current maternity care structures was a key motivation in the design of this review. Maternity care providers require guidance on the aspects of care that can be safely and effectively delivered via remote interventions and consider the implications in terms of acceptability, fidelity, and equitability [19-21]. The COVID-19 pandemic presented a further motivation for this review, considering the impact of the pandemic on women’s mental health and identifying innovative ways of delivering safe, effective, and equitable care [22,23]. In this review, we seek to identify the types of remotely delivered interventions that are available and have been evaluated to improve the symptoms of anxiety in women who are pregnant. We include a broad concept of common anxiety disorders in pregnancy, including pregnancy-specific anxiety and fear of birth, and a range of intervention strategies, including psychological, mind–body, education, and social support. The review aims to answer the following questions:

- What remotely delivered, digital, or web-based interventions have been evaluated and reported in the research literature to improve the symptoms of anxiety in women who are pregnant?
- How effective are remotely delivered, digital, or web-based interventions in reducing the symptoms of anxiety in pregnancy?
- What are women’s views, attitudes, and experiences of accessing and participating in remotely delivered anxiety interventions during pregnancy?

## Methods

### Overview

A mixed methods systematic review was conducted following the established Joanna Briggs Institute (JBI) methodological guidance [24]. This approach supported the broad research aim and enabled evidence from diverse study designs to provide a comprehensive and detailed understanding of the current evidence base [25,26]. The review adopted a convergent segregated mixed methods approach [27] in which the search and synthesis process for the different study designs included in the review were conducted concurrently. Qualitative and quantitative evidence were initially analyzed separately, followed by comparison and integration of qualitative and quantitative data to form an overarching synthesis. Before commencement, the review protocol was registered on the PROSPERO (The International Prospective Register of Systematic Reviews) database (CRD42020195887).

### Eligibility Criteria

Papers were included if the following criteria were met:

1. Participants included women who are pregnant of all parities across the 3 trimesters of pregnancy; women who are pregnant and under the care of specialist mental health services for severe and enduring mental health conditions were excluded
2. Evaluated the following types of interventions: mind–body interventions (relaxation, yoga, meditation, mindfulness, hypnotherapy, and imagery), social support interventions (supportive interactions, group discussions, peer support, telephone support, and exercise), educational interventions (birth preparation, educational sessions, educational materials, psychoeducational interventions, and antenatal classes), psychological interventions (CBT, mindfulness CBT, group CBT, interpersonal psychological therapy, nondirective counseling, therapy, and problem-solving therapy), and interventions that have been remotely delivered, including web-based materials, via telephone, computer software, digital apps, and digital forums
3. Comparators or control groups (CGs) included usual antenatal care or other types of interventions
4. Interventions were considered appropriate and capable of being introduced into maternity care
5. Outcome evaluations included measures of anxiety, fear, stress, distress, and women’s views, feedback, and opinions
6. Study methods included quantitative or qualitative primary research, including mixed methods studies involving any number of participants

### Information Sources and Search Strategy

A search of the following electronic bibliographic databases was undertaken (October to November 2020): the ACM Digital Library; Allied and Complementary Medicine Database; Applied Social Sciences Index and Abstracts; Centre for Reviews and Dissemination database; the Cochrane Central Register of Controlled Trials; the Cochrane Library; the Cochrane Depression, Anxiety and Neurosis Group’s Trials Register; CINAHL; Embase; Health Technology Assessment Library; IEEE Xplore; JBI; Maternity and Infant Care; MEDLINE; PsycINFO; and the Social Science Citation Index. References cited in existing systematic reviews and meta-analyses and reference lists of identified studies were searched to identify additional potentially relevant studies.

The search was limited to studies published since 2000 focusing on women who are pregnant and written in English. This reflects the period in which digitally delivered interventions were available in maternity care.

**Figure 1:**
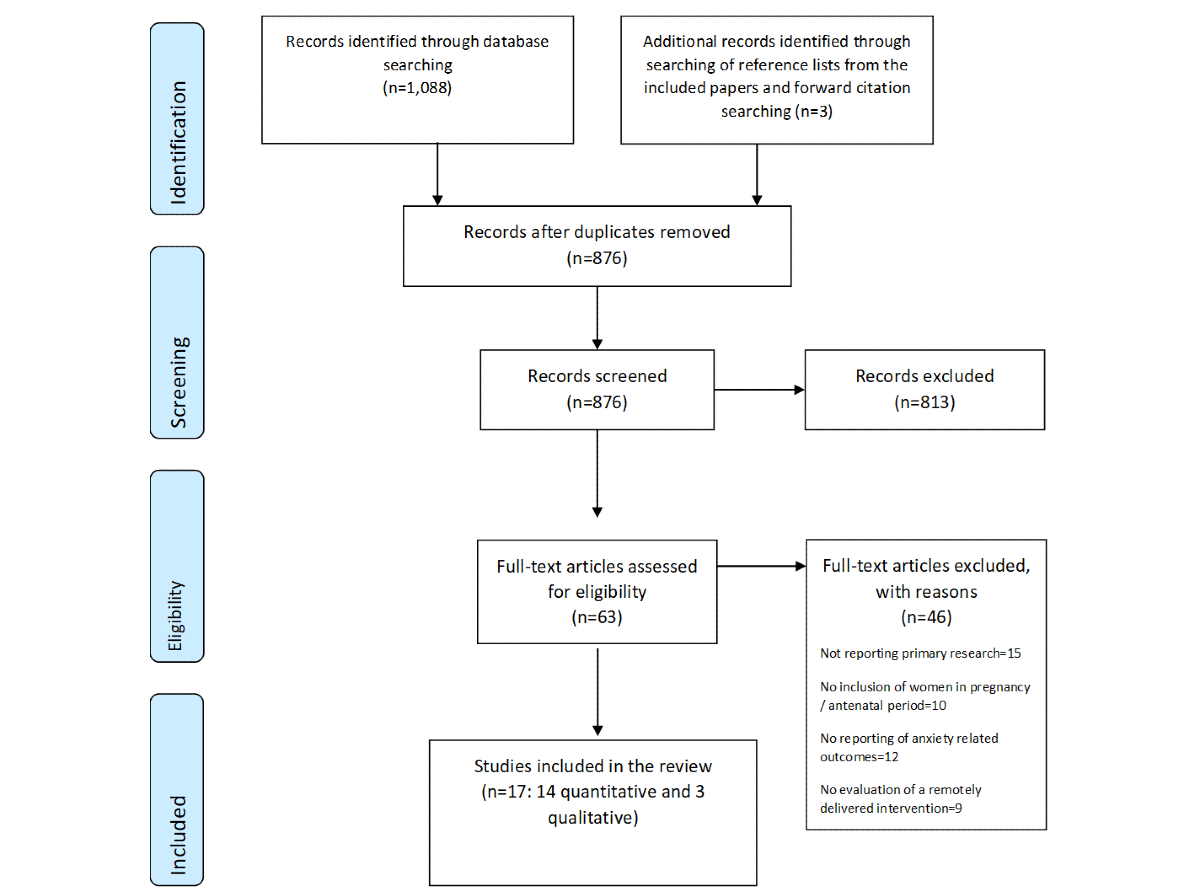
PRISMA (Preferred Reporting Item for Systematic Reviews and Meta-Analyses) diagram, remotely delivered interventions for anxiety in pregnancy.

Titles and abstracts of papers were screened independently by 2 reviewers (KE and SC) against the inclusion and exclusion criteria to identify potentially relevant papers. Potentially relevant papers were retrieved and read in full to identify papers for inclusion in the systematic review. The papers identified for inclusion were agreed upon following discussions with the entire review team. A PRISMA (Preferred Reporting Item for Systematic Reviews and Meta-Analyses) flowchart (Figure 1) [28] was completed to summarize the study selection process. Reference management (RevMan, Version 5.4; The Cochrane Collaboration, 2020) software was used to organize and catalog the references.

Search terms used in the review are summarized in Textbox 1.

**Summary of search terms.**

**Search terms**

- “exp Anxiety/”
- “Anxiety Disorders/”
- “Fear/”
- “worries.mp”
- “worry.mp”
- “anxiety.mp”
- “anxious.mp”
- “fear.mp”

AND

- “Computer assisted instruction/”
- “Software/”
- “Social Media/”
- “Computer Communication Networks/”
- “Internet/”
- “Telemedicine/Telehealth/”
- “Internet-based intervention/”
- “Online intervention/”
- “Mobile applications/”
- “Information services/”
- “Video games/”
- “Monitoring physiologic/”
- “digital.mp”
- “electronic.mp”
- “e-learning.mp”
- “mobile applications.mp”
- “web.mp”
- “web-based.mp”
- “virtual.mp”
- “telephone.mp”
- “skype.mp”
- “face-time.mp”
- “video.mp”
- “video conferenceing.mp”

AND

- “Pregnancy/”
- “Peripartum period/”
- “Perinatal Care/”
- “childbearning.mp”
- “ante*natal.mp”
- “ante*partum.mp”
- “pregnancy.mp”
- “peripartum.mp”
- “perinatal.mp”

AND

- “Clinical Trial/”
- “Randomized Controlled Trial/”
- “systematic review”/
- “Meta-analysis/”
- “Cohort studies/”
- “Non-randomised/ Prospective/”
- “Case-Control studies/”
- “Qualitative research/”
- “Research Design/”
- “cohort.mp”
- “rct.mp”
- “randomised controlled trial.mp”
- “Quasi-experimental.mp”
- “Intervention.mp”
- “Case-control.mp”
- “Focus groups/”
- “Interviews/”
- “User experience.mp”
- “Design research.mp”
- “Intervention.mp”

### Risk of Bias and Quality Assessment

The quantitative experimental study designs were independently assessed by 2 reviewers using the Cochrane Collaboration Effective Practice and Organization of Care tool for assessing the risk of bias [29]. This assesses the risk of selection, performance, detection, attrition, and reporting biases. The RevMan (version 5) software package was used to assist the organization and presentation of the data. The Critical Appraisal Skills Program for qualitative and cohort studies, the Critical Appraisal Skills Program for qualitative and cohort studies [30], The National Heart, Lung, and Blood Institute Quality Assessment Tool for Before–After (Pre–Post) Studies with No Control Group [31], and the JBI Checklist for Quasi-Experimental Studies [32] were also used to assist the methodological assessment of studies for inclusion in the systematic review analysis. Although no studies were excluded on the basis of quality, the quality assessment was used to identify the strengths and limitations of the review [24].

### Data Extraction

Data extraction forms were designed and piloted. Extraction was completed by 2 independent researchers (KE and SC). Data extraction tables comprising numerical and textual data were produced to present the study characteristics, results, and quality assessments.

### Data Synthesis

Tables were produced to present the study characteristics, results of interest, risk of bias ratings for randomized controlled trials (RCTs), and quality ratings for the included papers. This is presented alongside a narrative description of the data. A meta-analysis of quantitative data from the included experimental studies was conducted [29]. Considerations for performing meta-analysis included assessing the risk of bias of the studies and performing a qualitative assessment of clinical homogeneity. To evaluate statistical heterogeneity, the Q statistic and *I*^2^ index were used [33]. A random effects model was identified as the most appropriate method of analysis, and subgroup analysis was planned for subsets of intervention designs, participants, and methods of delivery. The standardized mean difference (SMD) was presented as the summary statistic for continuous data for anxiety and other outcomes, with 95% CIs and 2-sided *P* values calculated for each outcome where possible. A Grading of Recommendations Assessment, Development and Evaluation (GRADE) assessment [29,34] of the quantitative review findings was conducted, and a GRADE Confidence in Evidence from Reviews of Qualitative research (GRADE-CERQual) assessment of the qualitative studies [35] was planned, although there were insufficient data to perform a GRADE-CERQual assessment.

Following the convergent segregated approach [27], quantitative findings were translated into narrative statements that were then compared, contrasted, and juxtaposed against the qualitative findings and integrated to configure synthesized findings to address the research questions. The synthesized findings were then interpreted and configured into overarching themes to develop explanation, understanding, and coherence between the synthesized findings as a whole [26,36].

## Results

Details of the included qualitative and quantitative studies, population, intervention components, and delivery are presented in Multimedia Appendix 1 (TIDieR [Template for Intervention Description and Replication] checklist) [37].

### Quality Assessment

**Figure 2:**
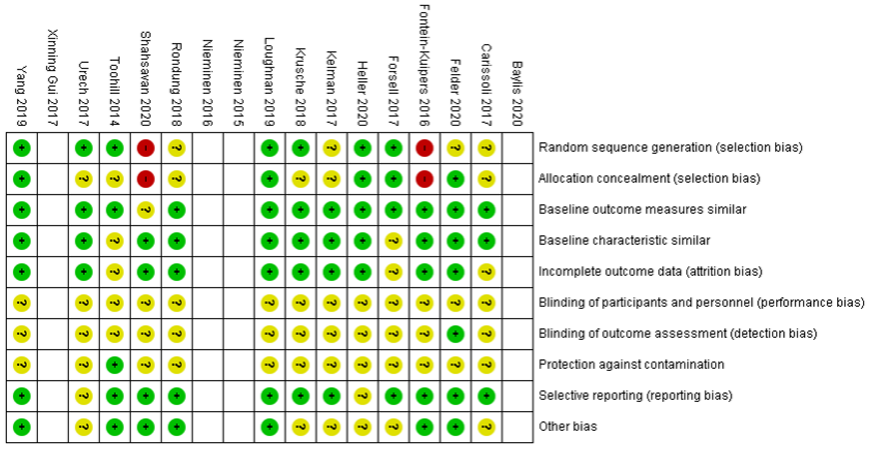
Risk of bias table with assessments of the experimental studies [16,17,38-41,43-47,49-52,62].

**Figure 3:**
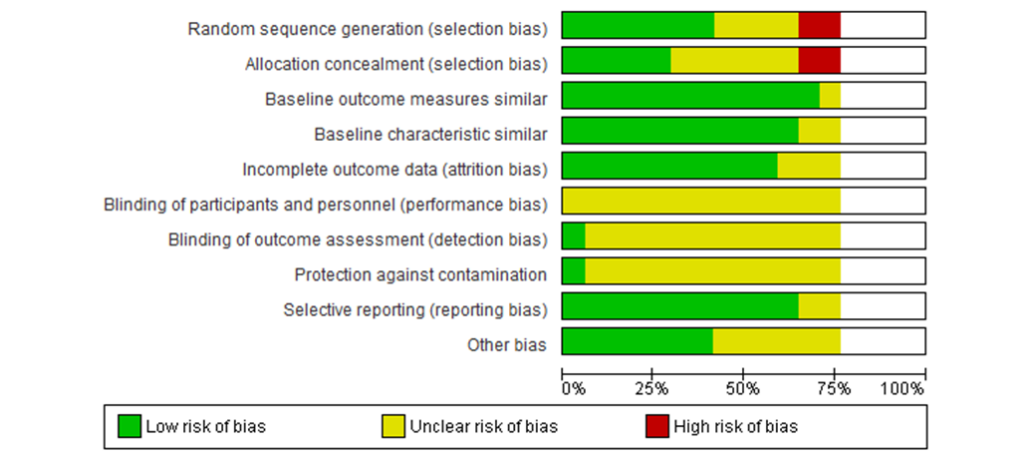
Risk of bias summary of the quantitative experimental selected studies.

The risk of bias assessments for quantitative experimental studies are presented in Figures 2 and 3. Studies were assessed as having a high or moderate risk of bias against the risk of bias standards for studies with a separate CG [29]. A cohort feasibility study [16] was assessed as moderate for methodological quality, which was mainly attributed to a lack of reporting of the recruitment strategy and the small sample size. The 3 qualitative studies were assessed using a moderate to high methodological quality score. Moderate scores were allocated to studies assessed as having limited reporting of (1) researchers’ reflexivity and (2) Sampling and context [38,39].

### Results From the Quantitative Studies

#### Overview

Of the 16 included studies, 14 (88%) were conducted in Australia, China, Iran, Italy, the Netherlands, Sweden, Switzerland, the United States, and the United Kingdom. Of the 14 studies, 10 (71%) were RCTs, 2 (14%) were quasi-experimental studies, 1 (7%) was a controlled study, and 1 (7%) was a cohort study. Participants ranged from 28 to 433, with a total of 2339 participants across all included quantitative studies. Study populations included a general population of women who were pregnant (no psychological screening criteria), women with high anxiety and depression self-report scores, women with high fear of childbirth, women with insomnia, and preterm labor.

#### Study Rationale

In all the included studies, the authors stated that the effectiveness of cognitive-based or mindfulness approaches had been demonstrated in wider populations using traditional face-to-face sessions, with some evidence of effectiveness for internet-based CBT (I-CBT) approaches for anxiety and depression. Studies that focused on supporting women with fear of birth reported on the associations among anxiety, stress, and fear of birth. The authors hypothesized that CBT-based interventions would also lead to an improvement in women’s fear of birth symptoms. Approximately 14% (2/14) of studies were focused on addressing birth outcomes. Urech et al [40] discussed the association between anxiety and preterm birth, suggesting psychological therapy as potentially helpful in improving birth outcomes. Shahsavan et al [41] reported an association between maternal requests for cesarean section and fear of birth, stating that addressing fear of birth symptoms may lead to a reduction in the prevalence of cesarean birth. Many of the included studies provided a rationale for developing remotely delivered interventions related to (1) flexibility, (2) cost-effectiveness, (3) potential reach, (4) accessibility, (5) availability, and (6) ubiquity. The flexibility of remotely delivered interventions was considered particularly relevant during pregnancy. The authors suggested that barriers to accessing traditional treatment, such as childcare issues, access to transportation, and limited time, could be overcome by providing interventions that women could access at the time of their choosing. Many studies have reported a lack of suitably trained therapists to meet the demand for psychological services, highlighting the accessibility of I-CBT as an advantage for increasing access to care for all women in all locations. Approximately 14% (2/14) of studies [17,42] discussed that remotely delivered interventions might provide women with greater anonymity and be more acceptable to women who may be reluctant to disclose their symptoms or seek support from health care professionals (HCPs) because of the stigma of mental health conditions in pregnancy, providing women with greater anonymity. The propensity of women who are pregnant for accessing web-based information and the ubiquity of smartphones, tablets, and computers was highlighted as further motivation for developing remotely delivered interventions for this population.

#### Comparators or CGs

Carissoli et al [43] conducted a pragmatic controlled trial, with the intervention group (IG) receiving a psychological well-being mobile app in addition to childbirth classes. The CG was randomized to receive only childbirth classes. Felder et al [44] recruited women with symptoms of insomnia. The CG was reported to receive standard care for insomnia, which the authors state may have included medication, psychotherapy, herbal supplements, counseling, and support groups. Urech et al [40] recruited women with diagnosed preterm labor for a cognitive behavioral stress management program. The CG received the same duration of web-based sessions and were asked to write short stories, listen to radio plays, answer specific questions, or perform quiz games. Other psychological, psychoeducation, and mindfulness intervention studies stated standard or usual antenatal care as the control condition [17,41,42,45-48,52]. The study by Rondung et al [49] compared I-CBT for women with fear of birth with standard care, which the authors described as including midwife-led fear of birth counseling, delivered face-to-face over 2 to 4 sessions. Kelman et al [50] compared internet-delivered compassionate mind training with I-CBT in a proof-of-concept RCT.

#### Intervention Components in the Included Studies

Interventions included psychological (CBT and problem solving), mindfulness, and educational designs. Many of the interventions adopted a multidimensional approach with cognitive behavioral techniques, relaxation, and educational intervention components. Most were delivered via web-based platforms where women could complete exercises and modules in their own time. Of the 13 interventions, 8 (62%) interventions included direct contact from trained therapists or coaches via feedback or answering questions, and 1 (8%) intervention was delivered via telephone [46]. Interventions lasted for 2 to 30 weeks (Multimedia Appendix 2). Participants were recruited by various methods: HCP referral during routine clinical appointments or self-recruited via advertisements in print and social media. Eligibility screening was conducted in 79% (11/14) of the studies, which included anxiety symptoms, depression, fear of birth, insomnia, or preterm labor (Multimedia Appendix 3). For studies reporting inclusion rates (as a percentage of the enrolled population), all 29% (4/14) of studies that used criteria based on fear of birth measures reported inclusion rates <10%. The 29% (4/14) of studies that used criteria based on anxiety or depression screening tools reported inclusion rates between 19% and 46%. Studies that did not apply psychological screening inclusion measures reported inclusion rates ranging from 76% to 90%.

#### Intervention Completion Rates Reported in the Included Studies

Shahsavan et al [41] reported an adherence rate of 93.72% across an 8-week I-CBT intervention. Adherence to the intervention program was measured as the number of log-ins and time spent on the designed software gathered via web‐logging per unique user ID. Loughnan et al [53] and Yang et al [45] reported completion rates of 72% across all 3 sessions for an I-CBT intervention and 84% for a mindfulness intervention. The remaining 54% (7/13) of interventions that provided relevant data reported falling attendance rates across the time frame of the intervention. Approximately 36% (5/14) of studies (I-CBT and problem-solving interventions) maintained completion rates of approximately ≥50% at week 5 to week 6 time points and included women with elevated symptoms of anxiety, depression, fear of birth, or insomnia [16,17,42,44,46]. Approximately 14% (2/14) of studies reported completion rates of <30% by week 3 on the intervention program: the study by Rondung et al [49], which involved I-CBT for women with high fear of birth scores, and the study by Krusche et al [47], which involved mindfulness for a general population of women who are pregnant. Fontein-Kuipers et al [48] reported the number of women who completed third trimester postintervention measures for an intervention delivering coping skills, resources, and personalized feedback (CG 56% and IG 64%). Across the 64% (9/14) of studies reporting full intervention completion data, all 56% (5/9) of studies that maintained completion rates >50% across the whole intervention reported significant improvement in anxiety scores between the IG and CG groups [16,41,44,46,53]. The 44% (4/9) of studies where completion fell <50% (at weeks 2-5) reported nonsignificant differences between the IG and CG groups [17,42,47,49].

#### Reported Acceptability and Satisfaction

Approximately 29% (4/14) of the studies assessed participant acceptability or satisfaction through feedback and satisfaction questionnaires; all received positive feedback, and women considered they had benefitted from participating. Loughnan et al [52] reported that after 1 to 2 sessions, most participants found the I-CBT intervention logical and considered it would be useful for their symptoms of anxiety and depression. Following completion of the intervention, most women were satisfied with the program, which they assessed as good quality, relatable, and useful in helping them manage their symptoms. Women could relate to the fictional characters experiencing anxiety and depression presented in an illustrated story. The authors reported that just over half of the participants preferred the web-based delivery of the program rather than other methods of intervention delivery. Forsell et al [17] reported that women who completed the I-CBT intervention felt satisfied, and most rated the treatment as helpful and important. Heller et al [42] reported that most women found the guided problem-solving intervention satisfying and would recommend the intervention to others. The website and feedback of coaches were rated as fairly good to excellent by most participants. The mindfulness intervention by Yang et al [45] was reported as beneficial by most of the participants who reported that it helped them feel relaxed and calm, become fully aware of fetal movements, and relieve discomfort. Some women reported that learning to maintain an accepting attitude was challenging, and daily mindfulness practice was onerous.

#### Reported Findings for Anxiety and Fear of Birth Scores in the Included Studies

Studies used various self-report outcome measures for anxiety and fear of birth symptoms, including the following:

- GAD 7-item (GAD-7) scale [18]
- Patient Health Questionnaire 9 item [54]
- Hospital Anxiety and Depression Scale—Anxiety subscale [55]
- Depression Anxiety Stress Scale [56]
- State–Trait Anxiety Inventory [57]
- Pregnancy-Related Anxiety Test [58]
- Pregnancy-Related Anxiety Questionnaire [59]
- Wijma Delivery Expectancy/Experience Questionnaire [60]
- Fear of Birth Scale [61]

Outcome scores as reported in the included papers are provided in Multimedia Appendix 3.

#### Meta-analysis of Anxiety Postintervention Scores

**Figure 4:**
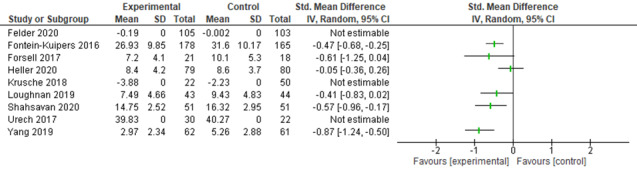
Studies reporting digitally delivered interventions for intervention and control groups (anxiety outcomes) [17,40,41,43,45,46,49,50,53].

**Figure 5:**
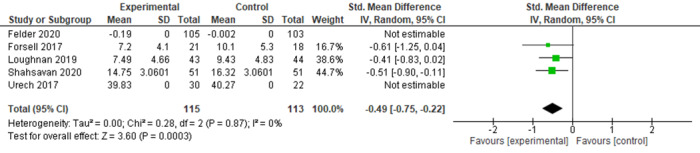
Studies reporting digitally delivered cognitive behavioral therapy interventions for intervention and control groups (anxiety outcomes) [17,40,41,45,52].

**Figure 6:**
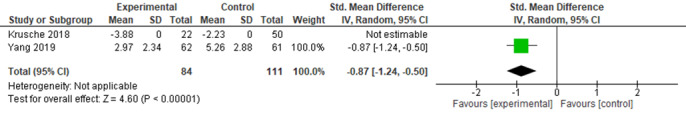
Studies reporting digitally delivered interventions with facilitator or therapist support for intervention and control groups (anxiety outcomes) [17,40,41,43,46,50].

Studies used different anxiety measures to assess intervention outcomes (GAD-7, Hospital Anxiety and Depression Scale—Anxiety subscale, Pregnancy-Related Anxiety Test, Pregnancy-Related Anxiety Questionnaire, Patient Health Questionnaire-9 item, State–Trait Anxiety Inventory, and Depression Anxiety Stress Scale); therefore, SMDs were used as the summary statistic [33]. The results from 43% (6/14) of the studies that provided sufficient outcome data for postintervention anxiety scores were pooled and indicated statistical heterogeneity among the studies and clinical heterogeneity between the intervention type, duration, and the characteristics of participants (Figure 4). Subgroup analyses were conducted on studies of interventions with similar characteristics, such as I-CBT and facilitated interventions, which provided sufficient data and were assessed as having sufficient clinical and statistical homogeneity to perform a meta-analysis (Figures 5 and 6) [33].

For the 23% (3/13) I-CBT interventions that provided sufficient data to be included in the meta-analysis, a beneficial effect was observed in relation to the reduction of anxiety scores (SMD −0.49, 95% CI −0.75 to −0.22), with low statistical heterogeneity among the studies (*I*^2^=0%; *P*=.87). For facilitated interventions, beneficial effects were observed in relation to the reduction of anxiety scores (SMD −0.48, 95% CI −0.75 to −0.22), although there was substantial statistical heterogeneity among the studies (*I*^2^=65%; *P*=.02). However, the pooled number of participants was relatively small (n=228 and n=848, respectively), and studies were assessed to have an unclear risk of bias. Therefore, the results of the meta-analysis should be interpreted with caution (Table 1).

**Table 1:** GRADE^a^ quality of evidence summary for I-CBT^b^ and mindfulness RCTs^c^ and quasi-experimental studies with anxiety score outcomes.

Interventions	Number of participants included in the analysis (N)	Quality of evidence (GRADE)	Intervention versus comparator mean difference (95% CI)
I-CBT interventions	228 from 2 RCTs and 1 quasi-experimental study	Very low because of small sample sizes, moderate risk of bias, differences in the population inclusion criteria, and intervention components	−0.49 (95% CI −0.75 to −0.22) improved anxiety scores in the intervention group
Facilitated interventions	818 from 3 RCTs and 2 quasi-experimental studies	Very low because of small sample sizes, moderate risk of bias, differences in the intervention types, population inclusion criteria, and intervention components	−0.48 (95% CI −0.75 to −0.22) improved anxiety scores in the intervention group

^a^GRADE: Grading of Recommendations Assessment, Development and Evaluation.

^b^I-CBT: internet-based cognitive behavioral therapy.

^c^RCT: randomized controlled trial.

### Findings From the Qualitative Studies: Women’s Engagement and Experiences of Interventions

A total of 3 qualitative studies were included in this review. Of the 3 studies, 2 (67%) included participants of experimental intervention studies [38,39], and 1 (33%) reported women’s narratives from a web-based forum [51]. The study by Gui et al [51] was included in the review as the web-based forum was considered by the review team to fit with the eligibility criteria of an intervention, and the study provided useful and relevant data on women’s concerns, anxiety, stress, and fear during pregnancy.

Baylis et al [39] interviewed women (n=19) who had participated in the I-CBT intervention to support women with a fear of childbirth [49]. The women were interviewed in the postnatal period. The study aimed to describe women’s experiences of guided I-CBT for fear of childbirth and describe the content of that fear. The authors reported that women’s fears were focused on losing control in labor, fear for their life, or the health of the baby. I-CBT was reported to offer a supportive and flexible treatment, although most women would have preferred CBT to be delivered face-to-face. Remote delivery made it difficult for women to maintain their motivation. The I-CBT intervention was not tailored to women’s specific situations or fears, and women could not always relate to the content within the sessions. However, women said it helped them to think in different ways about the imminent birth, understand their fear, work with their emotions, and cope with uncertainty. Learning the skills to *step outside* of themselves and observe what was going on helped women relieve their fears. The interactions with the psychologist through emails received positive comments and were described as supportive and encouraging. Individual contact made the treatment more personal, and the relationship with the psychologist was reported by some women as the most important part of the therapy. For some women, contact by email was preferable to meeting a stranger in an unfamiliar hospital setting and made it easier to disclose their fears. Other women would have preferred meeting the psychologist in person; this was thought to have resulted in women feeling more supported and motivated to complete the program and helped them create a warm and trusting relationship. Some women actively sought out alternative ways of addressing their fear of childbirth, including face-to-face midwife-led counseling, particularly when they needed to process previous traumatic birth experiences. Some women contacted a psychologist, attended antenatal classes, or attended antenatal yoga sessions.

Nieminen et al [38] conducted a thematic analysis of narratives completed by nulliparous women participating in an I-CBT intervention for fear of childbirth [16]. This study aimed to describe women’s expectations of childbirth before and after I-CBT. The authors reported six main themes: fear, self-confidence, coping, the partner, the staff, and the baby. Themes were identified within three domains: my own role, the role of others, and attitude toward the baby. Women reported that, following I-CBT, their complete focus on fear, as well as anxiety and hopelessness, was replaced by an expectation that reflected increased confidence. Women described positive and more realistic expectations regarding themselves, their partners, and the staff that would look after them in labor. Before I-CBT, women provided very few comments about the health of the baby in labor and were more focused on their anxiety and on surviving painful labor and delivery, which some women described as a *nightmare*. Following I-CBT, women still reported uncertainty about the outcome of labor and birth; however, their attitude toward their fear had changed to become more manageable and less isolating. They developed self-confidence and strategies to cope with pain and seek support from partners and care providers.

The study by Gui et al [51] analyzed narratives from women who were pregnant and posting within a web-based community across the 3 trimesters of pregnancy. The study reported women’s motivations for seeking web-based support, which surrounded (1) having limited access to care providers (delay in accessing advice); (2) wanting to access alternative sources of advice and information; (3) having little social support; (4) experiencing conflict between pregnancy information and their own experiences; and (5) seeking general advice, reassurance, and emotional support. The authors reported that across all 3 trimesters, women who were pregnant closely monitored their symptoms, fetal movements, body shape, and test results. Women reported emotional stress about maintaining their pregnancy, adjusting their lifestyle, managing medication for mental health conditions, and managing family relationships. Participation in web-based peer support provided a strategy for women to manage their stress and anxiety. Emotional stress remained a concern for women in the third trimester, which is mainly related to fear and anxiety about the upcoming birth, personal relationships with family members with regard to preparing for the birth, and extreme physical discomfort because of their progressing pregnancy. The authors stated that women’s motivations for seeking web-based support were greater for women with particular symptoms who needed to access information immediately. The number of posts that contained statements about emotional stress was slightly larger than the number of posts that directly asked for emotional support, which the authors concluded was because of differences in the way women cope with stress (ie, some women actively seek social support, whereas others prefer to vent their feelings).

### Synthesis of Quantitative and Qualitative Evidence

The evidence from the quantitative studies (14/16, 88%) and the qualitative studies (3/16, 18%) were synthesized to draw overall conclusions and provide insight to guide the design of future studies. The synthesis findings are presented in Textbox 2 and relate to the research questions.

**Synthesis of the qualitative and quantitative evidence.**

**Quantitative, qualitative, and synthesized evidence**

**How effective are remotely delivered interventions in reducing symptoms of anxiety?**

- Quantitative evidence: There was limited evidence of effectiveness for cognitive behavioral therapy and facilitated interventions for women with symptoms of depression, anxiety, and fear of birth. Interventions may be more effective when interventions are targeted at women with psychological symptoms; interventions include facilitation and contact with therapists, health care professionals, or peer communities; completion rates are sustained above 50% across all sessions; and interventions include cognitive skills and relaxation or meditation.
- Qualitative evidence: There was limited evidence from 2 studies to suggest that internet-based cognitive behavioral therapy interventions can be beneficial in helping women develop coping strategies for dealing with uncertainties and accessing support from partners and health care professionals. Limitations of remotely delivered interventions include a lack of tailoring to specific fears and anxieties; a lack of motivation and encouragement to complete modules; an inability to form secure, trusting professional relationships; content is perceived as onerous.
- Synthesized findings: Overall, there is limited evidence to suggest that interventions are more effective when women are motivated to maintain regular participation in interventions. Strategies to maintain participation include the following:
  - Personalization: achieved by providing regular contact with therapists and health care professionals to discuss individual circumstances, symptoms, or concerns; consider animated therapists as a proxy for human therapist interactions
  - Relatable: designing and targeting interventions to women with particular symptoms; providing peer forums; presenting content as stories and vignettes
  - Skills and techniques: include components of relaxation and cognitive-based skills
  - Achievable: sufficient sessions to develop new skills without being too time consuming

**What are women’s views on remotely delivered interventions in reducing the symptoms of anxiety?**

- Quantitative evidence: There is limited evidence to suggest that attrition rates were higher in studies that did not include therapist and health care professional or peer support components and were not targeted at women with symptoms of anxiety, depression, or fear of birth. Participants in 2 internet-based cognitive behavioral therapy interventions reported they were satisfied with the intervention and considered it helpful to manage their symptoms.
- Qualitative evidence: There was limited evidence from 2 studies to suggest that internet-based cognitive behavioral therapy interventions for fear of birth were valued by women if they offered flexibility and strategies for coping with their anxiety and fear and provided access to individual support from therapists. Women with fear of birth would have preferred individualized advice that was not readily available via remotely delivered therapy. Although some women felt more confident in disclosing their fears via remote messaging, many women would have preferred and actively sought alternative face-to-face treatment.
- Synthesized findings: Overall, there is very limited evidence to suggest that some women may prefer face-to-face therapy to help them stay motivated and receive individualized, tailored advice to manage their fears surrounding pregnancy and childbirth. However, for other women, remotely delivered interventions that provide some contact with a therapist, health care professional, or peer community may provide adequate human interaction to be of benefit. Remote interventions may also provide women with greater anonymity to help them feel more confident in disclosing their symptoms. There is limited evidence from the qualitative and quantitative evidence to suggest that women value interventions that resonate with their experiences. Women may be more motivated to complete interventions that are perceived as relevant to their needs and situations.

## Discussion

### Principal Findings

This paper presents a systematic review of remotely delivered interventions to improve symptoms of anxiety in women who are pregnant and presents a meta-analysis of the preliminary efficacy of these interventions on self-reported anxiety scores. Within the 16 included studies, numerous intervention designs were evaluated. Of the 16 studies, 10 (63%) evaluated I-CBT interventions targeting women with fear of birth, anxiety and depression, preterm birth, and insomnia; 2 (13%) reported mindfulness interventions for women who are pregnant and having symptoms of anxiety and depression; and the remaining 4 (25%) included psychoeducation for women with fear of birth, problem solving for women with anxiety and depression, psychological well-being, and a web-based forum for a general population of women who are pregnant. Remotely delivered interventions included in the meta-analysis achieved some beneficial effect in relation to the reduction of anxiety scores, although these findings need to be interpreted with caution as sample sizes were relatively small, and studies were assessed to have an unclear risk of bias.

The need to increase the reach and improve timely access to therapeutic and supportive treatment for women with psychological symptoms was reported as the main rationale for conducting the studies. In the United Kingdom, suicide is the second largest cause of maternal deaths, and women with a mental health diagnosis are overrepresented among women who die during pregnancy or the postnatal period [62]. Within the UK maternity services, low-level psychological therapies are recommended as the first-line treatment option for women with mild to moderate mental health conditions [63]. Improving Access to Psychological Therapy programs are available for many women who are pregnant in the United Kingdom, with recommended treatment interventions starting within 6 weeks of referral [64]. Women have reported positive experiences of Improving Access to Psychological Therapy support, although barriers to accessing services have been identified, including reluctance to disclose difficulties because of stigma and fear of custody loss, as well as lack of clear information and support, with referrals from HCPs such as general practitioners, midwives, and health visitors [65]. This review has identified that for some women, remotely delivered interventions provide a sense of anonymity, which enables them to feel more confident in disclosing their symptoms. However, qualitative studies assessing women’s feedback on remotely delivered interventions reported a lack of face-to-face contact with therapists and a lack of tailoring information and interventions to women’s specific circumstances as barriers to the effectiveness of interventions. The quantitative data suggested that interventions were more effective in improving anxiety symptoms when interventions included individual contact from a therapist, HCP, or peer web-based forums.

Social learning and social comparison theory can be facilitated by providing social forums to foster a sense of connectivity and cooperation. Many women participate in web-based forums during pregnancy to discuss their worries and concerns and regularly access web-based information to monitor their well-being and assist in decision-making [51,66]. The inclusion of social support mechanisms as a component of remotely delivered interventions has been recommended for individuals with serious mental health concerns [67].

Therapeutic alliance has been reported as fundamental to the success of face-to-face psychological therapies and requires a strong bond between the care provider and client to foster shared understanding and collaboration on tasks and goals [67]. A systematic review of technology-based mental health interventions identified that users could experience therapeutic alliances with digital interventions if they are personalized and interactive, providing automated feedback that emulates reciprocal trusted relationships [68]. Numerous systematic reviews have identified that remotely delivered anxiety interventions are more effective when therapists provide support and guidance [69-71]. The impact of facilitator or therapist training has mainly been evaluated in digital interventions for symptoms of depression. Remotely delivered guided interventions were reported to be beneficial in improving symptoms of depression and found no association between the qualifications or level of facilitator training and outcomes [72-74]. In pregnancy, increasing midwives’ and maternity care providers’ awareness of remotely delivered interventions could assist signposting women to effective interventions. Remotely delivered interventions can be enhanced by midwives offering support and encouragement to improve intervention uptake and completion and providing pregnancy-specific information and advice tailored to women’s particular circumstances. Midwives may need brief, additional training to support this role; however, in addition to supporting women’s experience of the intervention, midwives’ involvement would reflect contemporary policy drivers related to the continuity of carer (Better Births). A central tenet of the Midwifery Continuity of Carer models is the development of a collaborative relationship and provision of personalized care. Compared with conventional care, the provision of continuity of carers enables midwives to get to know women better, increase mutual trust, and facilitate access to specialist services.

Of the 16 included studies [44,53], 2 (13%) evaluated unguided I-CBT interventions for women with anxiety and depression and women with insomnia. Both studies reported an improvement in postintervention between-group anxiety symptoms for the IGs. Both studies included fictional characters in the form of a digital therapist [44] and fictional characters to narrate anxiety and depression experiences and help teach CBT skills. Rehm et al [75] reported that avatars were used as autonomous digital therapists to facilitate clinical interviews and assessments, provide psychoeducation, or signpost to other services. Digital therapists, not controlled by human clinicians, can be represented as a realistic-looking human avatar or as a 2D animated character. Participants have reported that they were willing and felt comfortable sharing information with therapist avatars [75], although there is very limited evidence, and further research is required in the perinatal context.

None of the included studies were solely targeted at improving symptoms of anxiety, which may reflect a greater focus on broader concepts of psychological well-being and respond to the reported comorbidity and associations between common mental health and psychosocial and physical health concerns in the perinatal period [76-78]. Although a multidimensional approach has been reported as an important factor in promoting psychological well-being in pregnancy [79], interventions targeting 1 condition may not be effective for the other comorbid conditions [80]; the underpinning theory of change needs to be defined for each condition before further testing the mechanisms of change.

The 31% (5/16) of studies that maintained completion rates ≥50% across the entire intervention reported significant improvement in anxiety scores between the IG and CG groups compared with studies where completion rates fell <50% (weeks 2-5). Of the 4 studies where completion fell <50%, 3 (75%) were developed for the general population of women who are pregnant and 1 (25%) for women with severe symptoms of fear of birth. Studies that maintained relatively high completion rates were targeted at women with symptoms of anxiety, depression, insomnia, or fear of birth and used established, validated screening tools with recommended cutoff scores to identify women with mild, moderate, and severe symptoms. A systematic review of antenatal interventions to reduce maternal distress also reported that interventions delivered to women with symptoms of distress were more effective than preventative interventions for women with little or no symptoms at baseline [81]. However, because of the heterogeneity of target populations included in the review, there is currently insufficient evidence to draw conclusions about the effectiveness of targeted interventions compared with universal interventions for women with symptoms of anxiety during pregnancy.

Overall, the content of interventions was well-documented, with most of the studies reporting the material content provided in each module. Of the 13 interventions, 10 (77%) included cognitive skills, and 69% (11/16) of studies included mindfulness or relaxation techniques. The amount of time taken to complete modules was not well-documented, and some authors reported the average time women accessed websites and portals, whereas other authors reported the amount and types of information provided. Women reported that intervention content and homework exercises felt too onerous at times, and they found it difficult to remain motivated. In future studies, assessing and reporting the amount of time spent completing intervention modules would help inform optimal intervention content. Overall, the information about the ways in which interventions were developed and tailored to the population was limited, and many authors simply stated that interventions were amended from traditional CBT interventions to meet the needs of a pregnant population or to fit within the timescale of pregnancy. Conducting a needs assessment in the target population is an essential stage in intervention development to improve potential effectiveness [82]. O’Mahen et al [83] explored women’s needs to inform the modification of CBT for perinatal depression. The authors reported that an increased focus on interpersonal strategies is required to help women seek out normalizing information that counters rigid beliefs about motherhood from other mothers. Considerations of women’s ethnicity and socioeconomic status are also required to address particular worries and concerns and improve the relevance of the intervention content. Similar studies are required to inform the tailoring of interventions for women with symptoms of anxiety during pregnancy. Only 19% (3/16) of the included studies provided demographic data relating to the ethnicity of participants, and from these studies, most of the participants were White. The recent MBRRACE-UK: Saving Lives, Improving Mothers' Care report has highlighted inequalities in maternity care, with Black and Black British and Asian and Asian British babies up to twice as likely to be stillborn or die neonatally, suggesting that safety initiatives are failing to reach many women from higher risk ethnicities [84]. A low rate of participation among ethnic minority groups reduces the generalizability of mental health research findings, affects the development of effective interventions, and further widens health inequalities. Urgent attention is required to address inequalities in mental health provision for Black, Asian, and minority ethnic women, and researchers need to develop relevant, tailored interventions and recruitment strategies that reflect the diversity of the population.

### Limitations

This review adopted a broad approach, including different types of interventions for different populations of women who are pregnant. This limited the utility of the study findings as different measurement instruments were used by the authors, and there was insufficient data to calculate outcome scores across all included studies. As a meta-analysis of postintervention anxiety scores was only achievable for a small subgroup of studies, the aim of the study to assess the effectiveness of interventions was only partially achieved. However, low-powered analysis based on a small number of studies can provide useful insights by exploring interesting relationships that may emerge and highlighting deficiencies in a topic that requires further attention [85]. The overall quality of the included studies was rated as moderate; the small sample sizes and heterogeneity of interventions and study populations resulted in overall low quality of evidence for the quantitative studies. There were insufficient qualitative studies to conduct a narrative synthesis, which further limited the qualitative findings and the synthesis of qualitative and quantitative evidence. We did not locate any studies that focused solely on anxiety symptoms in pregnancy, and most quantitative studies were not powered to detect significant changes in anxiety scores. Studies not published in English were excluded from this review.

### Conclusions

The introduction of remotely delivered interventions has the potential to improve symptoms of anxiety in women who are pregnant. The results of the review are limited and need to be interpreted with caution, as the findings of the review were predominantly based on small sample sizes with heterogeneity between intervention designs, delivery, and sample populations. The synthesized findings highlighted components of interventions that may improve the effectiveness and acceptability of remotely delivered interventions. Most women valued individual web-based contact from a therapist or HCP to maintain motivation and access individualized information. There was some evidence of effectiveness for interventions that provided some form of facilitation and access to peer support. Overall, there was limited evidence to suggest that interventions are more effective when women are motivated to maintain regular participation. Interventions targeting women with psychological symptoms were more likely to maintain participation across the intervention time frame and report improvements in anxiety scores. Although there was some limited evidence of the benefits of remotely delivered interventions described as CBT or mindfulness, most studies included a multicomponent approach and provided cognitive and mind–body content. Most women reported satisfaction with the interventions and provided positive feedback. Some women may prefer and actively seek face-to-face therapeutic interventions. However, for some women, remotely delivered interventions provide women with greater anonymity to help them feel more confident in disclosing their symptoms. Interventions provided a timely and flexible approach and provided women with strategies for coping with their symptoms. Future research is required to identify ways that interventions can be tailored to meet the particular needs of diverse populations of women who are pregnant to improve their relevance and ensure equitable access. Researchers need to consider the structures of maternity care in which interventions are implemented to assist in signposting women and need to maximize the potential for maternity care professionals to provide encouragement, support, and motivation, enhancing the digital therapeutic approach.

## Appendix

Multimedia Appendix 1 Summary of the intervention studies included in the review: TIDieR (Template for Intervention Description and Replication) checklist (Hoffmann et al [37]).

Multimedia Appendix 2 Intervention components in the included studies.

Multimedia Appendix 3 Recruitment methods, inclusion and completion rates, and outcome measures reported in the included studies.

## Abbreviations

CBT: cognitive behavioral therapyCG: control group
GAD: Generalized Anxiety Disorder
GAD-7: Generalized Anxiety Disorder 7 items
GRADE: Grading of Recommendations Assessment, Development, and Evaluation
GRADE-CERQual: GRADE Confidence in Evidence from Reviews of Qualitative research
HCP: health care professional
I-CBT: internet-based cognitive behavioral therapy
IG: intervention group
JBI: Joanna Briggs Institute
PRISMA: Preferred Reporting Item for Systematic Reviews and Meta-Analyses
PROSPERO: The International Prospective Register of Systematic Reviews
RCT: randomized controlled trial
SMD: standardized mean difference
TIDieR: Template for Intervention Description and Replication
