# Supplementary material for: Remotely Delivered Interventions to Support Women With Symptoms of Anxiety in Pregnancy: Mixed Methods Systematic Review and Meta-analysis
Source: J Med Internet Res. 2022 Feb 15;24(2):e28093. doi: 10.2196/28093 (PMC8889484; doi:10.2196/28093)
Supplement: Multimedia Appendix 1 [file jmir_v24i2e28093_app1.docx]

| Multimedia Appendix 1: Summary of the intervention studies included in the review: TIDieR checklist (Hoffmann et al., 2014) | | | | | | | | | | |
| --- | --- | --- | --- | --- | --- | --- | --- | --- | --- | --- |
| Author / Year / Country of study | Brief name | Why | Who | What (components / materials / procedures) | How delivered | When and how much | Tailoring | Intervention engagement and adherence | Summary of findings **(anxiety scores)** |  |
| **Randomised controlled trials** | | | | | | | | | | |
| Forsell 2017  Sweden | I-CBT antenatal depression | Antenatal depression associated with negative health outcomes requires early identification and treatment. Individual face-to-face CBT found to be effective. Barriers to access include poor HCP knowledge of services, stigma, lack of time, transport, relevance to pregnancy. I-CBT may overcome barriers | Women with symptoms of major depression (SCID-I MADRS-S 15-35)  **N=42** | Reading material (75,000 words)  Assessments  Homework  Worksheets  CBT trained therapist providing regular feedback through written messages | Online platform | Women recruited at 10-28 weeks gestation.  **10-weeks**  Women logged on ave. 34.1 times (SD = 15.7). Sent 14.1 messages to therapist (SD = 7.8). Therapist spent ave. 2 h 30 min per patient over 10 weeks. | ICBT for depression – adapted for pregnancy:  Depression and relation to pregnancy. Focus on sense making and de-stigmatization, contrast between societal norms v expectations. Relationships | Completed ave. 5.3 modules (SD = 2.5). 82% received six or more modules. | ICBT significantly lower levels of depressive symptoms post treatment (p<0.001, Hedges g =1.21).  **GAD-7 scores:**  **IG post 7.2 (4.1) CG post 10.1 (5.3) p=0.1**  . |  |
| Felder  2020  US | Digital CBT for insomnia in pregnancy. | Insomnia associated with increased risk of adverse outcomes. CBT recommended in pregnancy and for insomnia. Demand for face-to-face CBT exceeds availability. I-CBT offers flexible timely options. | Women with DSM-5 criteria for insomnia  **N=208** | Sleepio CBT-I  sleep restriction, stimulus control, cognitive therapy, relaxation techniques, sleep hygiene, education.  Tailored, automated self-help. | Online platform using an animated digital therapist.  Access to online community | Women recruited who were <29 weeks gestation.  **6 weekly sessions** | Not reported | 68 women (64.8%) completed all 6 of the sessions, taking a mean (SD) period of 7.97 (2.08) weeks to complete. | Digital CBT-I group reported greater improvements in insomnia symptom severity compared with control group.  **GAD-7: Post intervention effect sizes between groups IG -0.19 / CG -0.002. Post effect size between groups – 0.188 (95% CI - -0.26- -0.10). P=<0.001 d=- 0.42 small effect size** |  |
| Author / Year / Country of study | Brief name | Why | Who | What (components / materials / procedures) | How delivered | When and how much | Tailoring | Intervention engagement and adherence | Summary of findings **(anxiety scores)** |  |
| Heller 2020  Canada | Guided internet tool for treatment of anxiety and depression in pregnancy | Depression and anxiety associated with poor outcomes. CBT and IPT are effective for perinatal mental health. Barriers may be overcome via internet-based self-help interventions. | Women with depression or Anxiety symptoms CES-D >15 OR HADS-A >7  **N=159** | Problem solving treatment (PST)  3 steps:  Problem-solving: define the problem and generate solutions, systematic plan, carry out and evaluate the solution. | Online platform  Trained coaches: feedback via email. | Women recruited who were <30 weeks gestation.  5 weeks – 1 module per week | Adapted for pregnant women from the MamaKits online tool. | Study extended - stopped following Interim analysis.  IG: 47% completed all 5 modules, 63% at least three, 89% at least one | IG: symptoms decreased more than CG, but effect sizes were small-medium and statistically not significant. Trial terminated early for reasons of futility based on the interim analysis.  **HADS-A: IG post 8.4 (4.2) CG post 8.6 (3.7)** |  |
| Kelman 2020  US | Proof of concept: Brief Internet‐based CMT and CBT for perinatal / intending to become pregnant women | Depression impacts on mother and infant. Face‐to‐face care has not adequately addressed this concern due to difficulties in scaling these resources. Internet interventions show promise in filling this void. | Women who were intending pregnancy, were pregnant or up to 1 year postnatal.  **N=137** | 45 min didactic for CBT / CMT with CBT exercises / CMT audio meditations. | Online platform  Educational information and email | 2 weeks course | Adapted to match the length and depth of the course | Not reported | CMT and CBT demonstrated near equivalence in improving affect, self‐reassurance, self‐criticism, and self‐compassion, CMT showed superiority to CBT in reducing depression and anxiety. **Pregnant subgroup: anxiety scores were not significantly different between groups (p = .09)** |  |
| Krusche 2018  UK | Mindfulness for pregnancy | Prenatal depression, stress and anxiety are predictors of PND and negative impact on the family. Helpful psychological interventions not available to all who need them. | Pregnant women self-referred  N=185 | 10 interactive mindfulness sessions body scan, mindful movement, breathing space, mindful eating. Videos and assignments. | Online platform | Women from 12 weeks gestation  4-week course (could start and stop as required). | Adapted from an 8-week course | Mean time to complete for those who did complete (n = 22) was 8.41 weeks.  IG = n=107 (81 lost to follow-up) 42% did not engage. | No group difference in stress from pre to post intervention.  The course was potentially beneficial for those who completed it, but levels of drop out were very high.  **GAD-7: MD IG -3.88 / MD CG -2.23 p=0.08** |  |
| Author / Year / Country of study | Brief name | Why | Who | What (components / materials / procedures) | How delivered | When and how much | Tailoring | Intervention engagement and adherence | Summary of findings **(anxiety scores)** |  |
| Loughnan  2019  Australia | MUMentum Pregnancy': Internet-delivered CBT for antenatal anxiety and depression | Anxiety and depression are common during pregnancy and associated with adverse outcomes for the mother and infant if left untreated. No studies have investigated iCBT for antenatal anxiety and depression. | Pregnant women: GAD / major depressive disorder PHQ-9 >9 GAD7 >9  **N=87** | Unguided I-CBT.  Psychoeducation, cognitive behavioural model, physical symptoms, self-care, relaxation, unhelpful thoughts and behaviours, accepting uncertainty, coping and problem-solving, activity planning, graded exposure, assertive communication, maintenance | Online platform | Women 13-30 weeks gestation  4-week period with 7 days lock out to space time before moving to next lesson. | Mumentum unguided ICBT adapted from clinician guided programme condensed over 3 sessions (from 6). | Approximately 60 min completing each lesson and 55 min revising and practising the skills learned.  Of the 36 women who started iCBT, 26 completed all three lessons of treatment (76% adherence rate) | **I-CBT produced moderate to large effect size reductions for anxiety on the GAD-**7 (Hedges’ g=0.76) and psychological distress (g=0.88). Only small nonsignificant differences were found for depression outcomes (g=<0.35). Participants reported that iCBT was an acceptable treatment for antenatal anxiety and/or depression. |  |
| Rondung  2018  Sweden | I-CBT for Women with Fear of Birth | Although many pregnant women report fear related to the approaching birth, no consensus exists on how fear of birth should be handled in clinical care. | Pregnant women with high fear of birth (FOBS scores >59)  N=325  CG had counselling with midwives as per standard care in the setting. | Written materials, audio files, photographs and assignments.  Understanding fear and anxiety; behavioural change; setting goals.  Emotion: physiological, cognitive and behavioural aspects; self-monitoring. Behaviour; learned and emotional; negative reinforcement. Cognition: automatic appraisals, childbirth related catastrophising. Mindfulness. Exposure: situational, imaginative; avoidance; related to childbirth. Relapse prevention, maintenance | Online platform | Women 17-20 weeks pregnant  8 modules  Time to complete modules 10 – 38 minutes.  Guided by psychologists (active x3 times per week) | Based on CBT emotional disorder face-to-face protocol adapted to meet needs of the population | IG: 81% (103/127) commenced treatment. Mean time logged in the portal was 39.96 minutes. 60 (47%) moved on to the second module, and only 13 (10%) finished ≥4 modules. | The levels of fear of birth did not differ between the intervention groups postintervention. At 1-year postpartum follow-up, participants in the guided ICBT group exhibited significantly lower levels of fear of birth (U=3674.00, z=−1.97, P=.049, Cohen d=0.28, 95% CI –0.01 to 0.57).  **Women with high FOB**  **IG pre 74.26 (5.73) Post 67.15 (8.62)**  **CG Pre 69.29 (7.33) Post 65.73 (9.65)** |  |
| Author / Year / Counry of study | Brief name | Why | Who | What (components / materials / procedures) | How delivered | When and how much | Tailoring | Intervention engagement and adherence | Summary of findings **(anxiety scores)** |  |
| Toohill  2014  Australia | A Telephone Psycho-Education Intervention by Midwives in Reducing Childbirth Fear in Pregnant Women | Although intervention studies have focused on improving vaginal birth rates, no trials have reported on the effectiveness of an antenatal intervention to reduce fear levels before birth | Women attending antenatal clinics. High childbirth fear (W-DEQ A >65)  **N = 339** | Review expectations and feelings around fear of childbirth, support the expression of feelings, work through distressing elements of childbirth. BELIEF intervention helps develop situational supports, affirming that negative things can be managed with a simple plan | Telephone | Second trimester of pregnancy.  Intervention delivered at 2 time points (24 and 36 weeks of pregnancy) | Adapted from a midwifery counselling framework for distressed postpartum  women developed by Gamble and Creedy (2009). | IG: 84% received the intervention (142/170).  Mean duration of the first session was 58 minutes (range = 22–125) and 45 minutes for the second session range = 10–104 minutes). | Significant differences between groups on postintervention fear of birth scores and childbirth self-efficacy  **Women with high FOB W-DEQ A**  **IG: mean change 19.52 (18.59) CG: mean change 9.28 (16.32) p<0.01. Effect size 0.59** |  |
| Yang  2019  China | Effects of an Online Mindfulness Intervention Focusing on Attention Monitoring and Acceptance in Pregnant Women | Anxiety and depression associated with adverse outcomes. Mindfulness programs have reported reduced rates of anxiety, depression, and stress during pregnancy and have improved self-efficacy. Internet-based mindfulness interventions, are more accessible. | Pregnant women with Mild -moderate anxiety and depression GAD-7 >4 / PHQ-9 >4 (excluded GAD7>14 / PHQ9 > 14)  **N=123** | Pre-recorded Mindfulness material with audio text and pictures – homework exercises | Digital mobile platform  On-line interaction group. Trained nurses and midwives interacted via telephone or video link | Women at 24-30 weeks gestation  8 weeks - 4 x 40 minutes sessions every 2 weeks  Mean time on each meditation 21.23 minutes (SD 16.16).  Mean meditations per week was 3.25 (SD 1.45) | Adapted for pregnancy, no further information on adaption reported | IG: 52 (83.9%) women completed at least 3 sessions. Adherence to daily mindfulness practice was low. | The retention rate and feedback suggested that the mindfulness intervention was feasible and acceptable among pregnant women. Participants in the IG showed greater declines in depressive and anxious symptoms compared with CG, as well a significant improvement in mindfulness skills.  **GAD-7: IG post 2.97 (2.34) CG post 5.26 (2.88) p=<0.001** |  |
| Urech  2017  Switzerland | Efficacy of an internet-based cognitive behavioural stress management training in women with idiopathic preterm labour (PTL) | Psychosocial and psychological factors (anxiety, depression and stress) have been linked to PTL. Pregnant women are found to be willing to participate in internet-supported perinatal interventions. | Pregnant women with diagnosed pre-term labour.  **N=93** | Psychoeducation - stress in pregnancy. Relaxation. Emotional training - pregnancy-related anxiety. Cognitive training, Problem solving and enjoyment. Psychoeducation coping with motherhood difficulties. | Online platform, email, on-line forum | Women at 18-32 weeks gestation  6 weekly sessions  Personal psychologist weekly feedback  Online forum | The content of the intervention was based on the stress management training adapted to meet the needs of pregnant women with PTL | Not reported | Birth outcome and psychological wellbeing did not differ between IB-CBSM and CG.  **STAI-S: IG Pre / post 48.7 / 39.83 CG Pre / post 50.27 / 40.27**  **STAI-T: IG Pre / post 43.97 / 38.03 CG pre / post 43.5 / 38.5**  **PRAT: IG Pre / post 2.36 / 2.02 CG Pre / post 2.45 / 2.10** |  |
| Author / Year / Country of study | Brief name | Why | Who | What (components / materials / procedures) | How delivered | When and how much | Tailoring | Intervention engagement and adherence | Summary of findings **(anxiety scores)** |  |
| Controlled study | | | | | | | | | | |
| Carissoli  2017  Italy | Enhancing psychological wellbeing of women approaching childbirth: A controlled study with a mobile application | Anxiety and depression associated with adverse outcomes. Wide range of interventions show promising results. Mobile applications show potential in managing stress and anxiety. | Health pregnant women attending antenatal classes.  N=78 | BenEssere Mamma self-help program for pregnancy wellbeing. Daily relaxation exercises and guided imagery exercises and an emotional awareness area, with a mood journal. | Android devices - app | Women in the third trimester  Suggested 5 practices over 4 weeks. | Not reported | Log files reported an irregular practice and lower use than suggested. | Women’s childbirth expectations improved in both groups. The only significant interaction between groups and pre-post comparison is related to the Autonomy scale.  **W-DEQ IG: Pre 27.97 (6.82) Post 26.76 (5.95) CG: Pre 28.76 (6.34) Post 27.09 (6.74)** |  |
| Quasi-experimental study | | | | | | | | | | |
| Fontein-Kuipers 2016  Netherlands | The effect of Wazzup Mama?! An antenatal intervention to prevent or reduce maternal distress in pregnancy | Developed WazzUp Mama?!, an intervention offered during midwife-led care to prevent or reduce maternal distress among healthy pregnant women. | Healthy pregnant women receiving midwifery-led care.  N=433 | Web-based tailored programme: 1) signs and symptoms of maternal distress 2) factors or situations which contribute to distress 3) management of distress. Personalised feedback based on screening including resources for self-management and local supportive services | Online platform | Women in the first trimester of pregnancy  30 weeks | Intervention developed for use in pregnancy | For the intervention group 80% completed time 1 measures and 65% completed time 2 measures. For the control group 73% completed time 1 measures and 56% completed time 2 measures | Control group: depression, anxiety, and pregnancy-related anxiety moved in the negative direction. Experimental group : scores moved in the positive direction.  **IG STAI pre 28.72 (9.58) Post 26.93 (9.85)**  **CG STAI pre 28.9 (9.38) Post 31.6 (10.17)**  **IG PRAQ pre 18.58 (7.01) Post 15.04 (6.42)**  **CG PRAQ pre 18.59 (7.07) Post 19.43 (7.17)** |  |
| Shasavan  2020  Iran | Internet-based guided self-help CBT on Iranian women's psychological symptoms and preferred method of childbirth | Application of Internet‐based guided self‐help CBT (I‐GSH‐CBT) interventions may be successful to decrease the caesarean prevalence rate by decreasing child-birth fear and other affective distress. | Nulliparous women with high CBF (score≥85 W-DEQ-A), depression and anxiety (DASS-42 10‐13/14),  **N=102** | Text, audio, video. Information, skills, and exercises: self‐monitoring, cognitive restructuring, relaxation, assertiveness, and problem‐solving. individuals received feedback from psychologists. | Software for mobile devices | Women at 30 weeks gestation  8 weeks | Not reported | The mean number of logins for the intervention group for 8 weeks showed that IPW had an adherence rate of 93.72% | Implementing the I‐GSH‐CBT significantly reduced CBF, DASS‐42 scores, and caesarean section preference.  **IG pre 89.51 (+-1.62) Post 84.81 (+-1.34)**  **CG Pre 86.96 (+-0.63) Post 90.19 (+-0.96)** |  |
| Author / Year / Country of study | Brief name | Why | Who | What (components / materials / procedures) | How delivered | When and how much | Tailoring | Intervention engagement and adherence | Summary of findings **(anxiety scores)** |  |
| Cohort study | | | | | | | | | | |
| Nieminen 2016  Sweden | Treatment of nulliparous women with severe fear of childbirth via the Internet: a feasibility study | Fear of childbirth associated with request for caesarean section. CBT effective for anxiety disorders, no studies on fear of childbirth. | Nulliparous pregnant women with a W-DEQ sum score ≥ 85.  **N=28** | Self-help manual CBT. Physiology of normal pregnancy, labour and delivery, possible complications. First trimester: psycho-education fear and anxiety. Second trimester goal setting. Third trimester tool to cope with physical reactions to fear – breathing and focusing. Labour preparations – challenge thoughts and feelings. Pain relief – exposure in vivo (pictures, films). Managing situations. Third stage – dealing with setbacks. Maintenance | Online platform | Women at 18-30 weeks gestation  8-week programme  Short, weekly individual feedback by a personal therapist. | Not reported | 15 participants followed all 8 weeks of CBT | A statistically significant (p<0.0005) decrease of FOC (W-DEQ sum score decreased pre- to post-therapy, with a large effect size.  **Pre W-DEQ compared with the last W-DEQ before delivery, statistically significant (p<0.0001) large within-group effect size (Cohen’s d = 0.95)** |  |
| Author / Year / Country of study | Brief name | Why | Who | What (components / materials / procedures) | How delivered | When and how much | Tailoring | **Authors conclusions** | |  |
| **Qualitative studies** | | | | | | | | | | |
| Baylis  2020  Sweden | Women’s experiences of internet-delivered iCBT for Fear of Birth | Fear of Birth is common in pregnant women and associated with negative physical and mental health. Clear comorbidity with anxiety and depression. I-CBT has been suggested as a treatment option | Women with FOB (FOBS >59) who participated in I-CBT RCT (* Rondung et al. 2018)    **N=19** | See Rondung et al. 2018 | Online platform | Interviewed postnatal 12-22 months (intervention delivered in pregnancy)  8 modules guided by a psychologist | Based on CBT emotional disorder face-to-face protocol adapted to meet needs of the population | Women’s descriptions of Fear of Birth varied. Most women would have preferred a face-to-face meeting. I-CBT for Fear of Birth may be an alternative for some women. | |  |
| Neiminen  2015  Sweden | Nulliparous pregnant women’s narratives of imminent childbirth before and after I-CBT for severe FOB | Fear of childbirth associated with request for caesarean section. CBT effective for anxiety disorders, no studies on fear of childbirth. | Nulliparous pregnant women with a W-DEQ sum score ≥ 85.  (*Neiminen et al. 2016)  **N=15** | See Neiminen et al. 2016 | Online platform | Thematic narratives pre / post I-CBT.  Women at 18-30 weeks gestation  8-week programme | Not reported | Following ICBT participants changed their attitude towards childbirth from negative to more positive. More realistic expectations regarding themselves, partner and staff | |  |
| Gui 2017  US | Support Seeking from Peers for Pregnancy in Online Health Community | A need to examine pregnancy as a unique staged condition and to develop a more holistic understanding of pregnancy in the context of computing research to understand the needs of pregnant women and how they seek support online. | Users of babycentre.com online community.  Pregnant women | Popular online forum (US, Brazil, India, UK, Canada and Mexico). ‘Mommy Mentor’ forums based on pregnancy trimesters – supportive place for pregnant women to get advice about pregnancy. | Online forum | 600 posts (200 for each trimester) and their associated comments were analysed for this study from a larger dataset. | Not applicable | Women were motivated to seek multiple types of support from peers due to limited access and help from HCPs, limited offline social support, and mismatch between online information and women’s actual experience. Online health communities serve as a platform for pregnant women to seek and receive both informal experiential and formal medical knowledge and advice from peers, as well as emotional support. However, the specific support needs of pregnant women vary across three gestational stages of pregnancy. | |  |
| **CBF** – Childbirth fear; CG– Control Group; **CMT** - compassionate mind training; **FOB** – Fear of birth; **HCP** – healthcare professional; **iCBT** – Internet delivered Cognitive Behaviour Therapy; **IG** – Intervention group; **RCT** – Randomised controlled trial  **DASS** - Depression Anxiety Stress Scale (Lovibond and Lovibond, 1995); **FOBS** - Fear of Birth Scale (Haines et al., 2011); **GAD-7** - Generalised Anxiety Disorder-7 item scale (Spitzer et al., 2006); **HAS-A** - Hospital Anxiety and Depression Scale – Anxiety subscale (Zigmond and Snaith, 1983); **PHQ-9** - Patient Health Questionnaire 9 item (Kroenke et al., 2001); **STAI** - State-Trait Anxiety Inventory (Spielberger et al., 1983); **PRAT** - Pregnancy Related Anxiety Test (Rini et al., 1999); PRAQ - Pregnancy Related Anxiety Questionnaire (Van den Bergh, 1990); **WDEQ** - Wijma Delivery Expectancy / Experience Questionnaire (Wijma et al., 1998) | | | | | | | | | |  |
