# Supplementary material for: Remotely Delivered Interventions to Support Women With Symptoms of Anxiety in Pregnancy: Mixed Methods Systematic Review and Meta-analysis
Source: J Med Internet Res. 2022 Feb 15;24(2):e28093. doi: 10.2196/28093 (PMC8889484; doi:10.2196/28093)
Supplement: Multimedia Appendix 2 [file jmir_v24i2e28093_app2.docx]

| Multimedia Appendix 2 : Intervention components in the included studies | | | | | | | | | | | | | | | |
| --- | --- | --- | --- | --- | --- | --- | --- | --- | --- | --- | --- | --- | --- | --- | --- |
| Author, intervention type | Kelman  I-CBT depression | Kelman  I-CMT depression | Carissoli  Psychological wellbeing in pregnancy | Krusche  Mindfulness depression, stress, anxiety | Loughnan  I-CBT depression or anxiety | Heller  Problem solving anxiety depression | Felder  I-CBT insomnia | Urech  I-CB stress management pre-term labour | Rondung  I-CBT fear of birth | Yang  Mindfulness depression and anxiety | Shasavan  I-CBT for fear of childbirth | Nieminen  I-CBT for fear of childbirth | Forsell  I-CBT depression | Toohill  Psycho-education Fear of childbirth | Fontein-Kuipers  Maternal distress, coping resources |
| Eligibility  Anxiety FOB |  |  |  |  | GAD7>9  PHQ9>9 | CES-D>15  HADS-A >7 | DSM insomnia | Preterm labour | FOBS>59 | GAD7>4-14  PHQ9>4-14 | W-DEQ>84  DASS42 10-14, | W-DEQ>84 | SCID depression | W-DEQ A >65 |  |
| Method of remote delivery | Internet  Email | Internet  Email | APP Android | Online course | Internet | Internet  Email | Online platform | Internet | Internet  Digital messaging | Mobile device | Mobile device | Internet | On-line platform / messaging | Telephone | Web based |
| One-to-one support |  |  |  |  |  | Trained coaches |  | Psychologist | Psychologists | Nurses and midwives | Psychologist | Therapist | CBT therapist | Midwives | Midwives |
| Materials | Information and exercises | Information, audio meditations | Exercises, mood journal | Exercises, information assignments | Illustrated story and  worksheets | Information, examples, homework | Animated therapist, diary | Not reported | Text, audio, photographs assignments | 40 min modules | Information, skills, and exercises | Information, questions, homework | Reading, assessments, worksheets | Listening and responding | Personalised feedback, coping resources |
| On-line forum |  |  |  |  |  |  | **✓** | **✓** |  | **✓** |  |  |  |  |  |
| Duration | 2 weeks | 2 weeks | 4 weeks  (20 exercises) | 4 weeks | 4 weeks  (3 lessons) | 5 weeks  (5 modules) | 6 weeks (6 modules) | 6 weeks  (6 sessions) | 8 modules | 8 weeks  (4 modules) | 8 weeks | 8 weeks  (8 modules) | 10 weeks  (10 modules) | 12 weeks (2 sessions) | 30 weeks |
| Psychoeducation |  |  |  |  | **✓** |  |  | **✓** | **✓** |  |  | **✓** | **✓** | **✓** | **✓** |
| Behavioural | **✓** | **✓** |  |  | **✓** |  |  |  | **✓** |  |  | **✓** | **✓** |  |  |
| Cognitive | **✓** |  | **✓** | **✓** | **✓** |  | **✓** | **✓** | **✓** |  | **✓** | **✓** | **✓** |  | **✓** |
| Relationships & assertiveness | **✓** |  |  |  | **✓** |  |  |  |  |  | **✓** | **✓** | **✓** |  |  |
| Imagery and exposure |  |  |  |  | **✓** |  |  |  | **✓** |  |  | **✓** |  |  |  |
| Monitoring and goal setting |  |  | **✓** | **✓** | **✓** |  |  |  | **✓** |  | **✓** |  |  |  |  |
| Coping, problem solving |  |  | **✓** | **✓** | **✓** | **✓** |  | **✓** |  |  | **✓** |  |  | **✓** | **✓** |
| Relapse prevention |  |  |  |  | **✓** |  |  |  | **✓** |  |  | **✓** | **✓** |  |  |
| Relaxation and sleep | **✓** | **✓** | **✓** | **✓** | **✓** |  | **✓** | **✓** |  |  |  | **✓** | **✓** |  | **✓** |
| Mindfulness |  |  | **✓** | **✓** |  |  |  | **✓** | **✓** | **✓** |  |  |  |  |  |
| Completion | Not reported | Not reported | Irregular, less than advised | 42% did not begin, 11% all 4 module | 26 / 36 72% all lessons | 47% all 5 modules, 63% 3 or > | 64.8% all 6 modules | Not reported | Only 10% completed ≥4 modules | 83.9% at least 3 sessions | adherence rate of 93.72% | 54% completed all weeks | 82% > 5 modules 9% all | 84% completed | 65% time 2 measures |
| Sig results between groups |  |  |  |  | **✓** |  | **✓** |  |  | **✓** | **✓** | **✓** |  | **✓** |  |
