# Supplementary material for: Remotely Delivered Interventions to Support Women With Symptoms of Anxiety in Pregnancy: Mixed Methods Systematic Review and Meta-analysis
Source: J Med Internet Res. 2022 Feb 15;24(2):e28093. doi: 10.2196/28093 (PMC8889484; doi:10.2196/28093)
Supplement: Multimedia Appendix 3 [file jmir_v24i2e28093_app3.docx]

|  | Multimedia appendix 3: Recruitment methods and number of participants, enrolled, randomised, completed and analysed | | | | | | | | | | | | | | |
| --- | --- | --- | --- | --- | --- | --- | --- | --- | --- | --- | --- | --- | --- | --- | --- |
| First author | | Forsell | Kelman | Loughnan | Heller | Yang | Krusche | Fontein-Kuipers | Carissoli | Felder | Urech | Rondung | Shasavan | Nieminen | Toohill |
| Country | | Sweden | US | Australia | Netherlands | China | UK | Netherlands | Italy | US | Switzerland | Sweden | Iran | Sweden | Australia |
| Target population | | Pregnant women with major depression | Pregnant women, pregnant within year, intending pregnancy | Pregnant women with anxiety and depression | Pregnant women with anxiety and depression | Pregnant women with anxiety and depression | Pregnant women | Pregnant women | Primiparous women | Pregnant women with insomnia | Pregnant women diagnosed with pre-term labour | Pregnant women with fear of birth | Pregnant women with high fear of childbirth | Pregnant women with severe fear of childbirth | Pregnant women with high fear of childbirth |
| Promotion methods | | Social media, websites, blogs, online forums, newspapers, pregnancy magazine  Posters flyers in clinics and psychiatry clinic | On-line recruitment system. Professional networks emails to HCPs. Participants were paid $2.50 - $5.00 in rounds of recruitment. | Social media websites, online forums and flyers distributed in maternity hospitals. | Newspapers, magazines, social media, pregnancy websites, patient’s associations. Information flyers and posters in maternity and clinics | Hospital clinic. Nurses distributed leaflets to women with mild-moderate symptoms of anxiety /depression | Email, social media. Pregnancy forums. Posters in community sites, schools, churches, shops | Midwifery practices | Childbirth class | Flyers in clinics, shops volunteer registry, social media. Electronic health messages and mail | Pregnancy  magazines and websites in Switzerland, Liechtenstein, Germany and Austria. Gynaecologists and midwives also recruited recruit patients. | Recruited at ultrasound scan appointment | Hospital clinics | Project home page | Antenatal clinics |
| Referral | | Self-referred | Self-referred | Self-referred | Self-referred | Referred by HCP | Self-referred | HCP recruited | Self-referred | Self-referred | HCP and self-recruited | HCP recruited | HCP recruited | Self-recruited | HCP recruited |
| Recruitment period | | Not reported | 6 months | 12 months | 3 years | 3 months | 12 months | 12 months | Not reported | 18 months | Not reported | 12 months | 4 months | 2 years | 12 months |
| Enrolled / assessed | | 95 | 153 | 409 | 349 | 642 | 237 | 567 | 78 | 2258 | 212 | 4502 | Not reported | Not reported | 4164 |
| Screening | | 1. MADRAS  Demographics  2. SCID MINI | Demographics | GAD7>9  PHQ9>9 | Demographics  CES-D >15 or HADS-A >7 | GAD 7 > 4  PHQ >4 |  |  |  | DSM – 5 Insomnia | Medically diagnosed pre-term labour | FOBS >59 | W-DEQ -A >84  DASS 10-14 anxiety | W-DEQ >84 | W-DEQ-A >65 |
| Randomised / Included (% of those enrolled) | | 42 (39%) | 137 (90%) | 87 (21%) | 159 (46%) | 123 (19%) | 185 (78%) | 433 (76%) | 78 (no screening) | 208 (9%) | 93 (44%) | 258 (6%) | 102 | 28 | 339 (8%) |
| Intervention completion rate | | 82% > 5 modules | Not reported | 72% completed all lessons | 47% completed all 5 modules  63% at least 3 | 83.9% at least 3 sessions. | 42% did not begin, 11% completed all 4 | Not reported | Not reported | 71% completed all 6 sessions | Not reported | Only 10% completed ≥4 modules. | 102 (100%) | 54% completed all weeks | 84% completed |
| Completion of post intervention measure IG / CG | | 95% / 90% | 59% CMT / 63% CBT | 68% / 92% | 68% / 81% |  | 17% / 25% | 65% / 56% | Irregular, less than advised | 95% / 97% | 55% completed psychological measures |  | adherence rate of 93.72% | 28 | 59% / 57% |
